# Association of subclinical myocardial injury with arterial stiffness in patients with type 2 diabetes mellitus

**DOI:** 10.1186/1475-2840-12-94

**Published:** 2013-06-22

**Authors:** Kai-Hang Yiu, Chun-Ting Zhao, Yan Chen, Chung-Wah Siu, Yap-Hang Chan, Kui-Kai Lau, Shasha Liu, Chu-Pak Lau, Hung-Fat Tse

**Affiliations:** 1 Division of Cardiology, Department of Medicine, the University of Hong Kong, Rm 1929b, Block K, Queen Mary Hospital, Hong Kong, China; 2 Research Centre of Heart, Brain, Hormone and Healthy Aging, Li Ka Shing Faculty of Medicine, the University of Hong Kong, Hong Kong, China

**Keywords:** Type 2 diabetes mellitus, Myocardial injury, Arterial stiffiness, High-sensitivity troponin I

## Abstract

**Objective:**

Type 2 diabetes mellitus (T2DM) is associated with subclinical myocardial injury although the underlying mechanism is uncertain. We postulated that arterial stiffness, endothelial dysfunction and subclinical atherosclerosis may contribute to subclinical myocardial injury in patients with T2DM.

**Methods:**

Serum high-sensitivity troponin I (hs-TNI) an indicator of myocardial injury, was measured in 100 patients with T2DM without clinical evidence of macrovascular disease and 150 age and gender-matched controls. Elevated hs-TnI was defined as follow (derived from the 99^th^ percentile from controls): Male >11.1 ng/L; female >7.6 ng/L. Measures that may contribute to myocardial damage in patients with T2DM, including brachial-ankle pulse wave velocity (ba-PWV), brachial flow mediated dilatation (FMD) and carotid intima media thickness (IMT), were also assessed.

**Results:**

The serum level of hs-TNI (5.7±9.2 μg/L vs. 3.2±1.9 μg/L, P< 0.01) and the prevalence of elevated hs-TNI (12% vs. 4%, P = 0.02) were significantly higher in patients with T2DM than controls. Patients with T2DM also had significantly worse ba-PWV (17.98±3.91ms^-1^ vs. 15.70±2.96 ms^-1^), brachial FMD (2.6±3.5% vs. 5.5±4.2%, P< 0.01) and carotid IMT (0.96±0.20 mm vs. 0.86±0.14 mm, P< 0.01). In patients with T2DM, hs-TNI was positively correlated with systolic blood pressure (r = 0.31, P<0.01), serum creatinine (r = 0.26, P = 0.01) and ba-PWV (r = 0.34, P< 0.01). Importantly, multiple regression revealed that only ba-PWV was independently associated with hs-TNI (β = 0.25, P = 0.04).

**Conclusion:**

The results demonstrated an independent association between ba-PWV and hs-TNI in patients with T2DM with no clinical evidence of macrovascular disease. These findings suggest that increased arterial stiffness is closely related to subclinical myocardial injury in patients with T2DM.

## Introduction

Patients with type 2 diabetes mellitus (T2DM) have up to a two-fold increased risk of cardiovascular morbidity and mortality [1,2]. As well as the close association with coronary artery disease, hyperglycemia can cause myocardial injury [3] and dysfunction [4], independent of underlying macrovascular disease.

While sophisticated cardiovascular imaging, such as cardiac magnetic resonance imaging, may detect subclinical myocardial injury, its clinical application is limited by its high cost and the need for a prolonged study time. Emerging biomarkers may provide an alternative means to non-invasively detect myocardial damage. High-sensitivity cardiac troponin levels, a novel marker that allows detection of troponin below clinical thresholds, has been shown to predict cardiovascular events in patients with T2DM [5], congestive heart failure [6] and the general population [7,8]. In addition to its prognostic value, high-sensitivity troponin can be used to detect subclinical myocardial injury in patients with T2DM with no overt macrovascular disease [3]. The underlying pathology of this subclinical myocardial injury in patients with T2DM has nonetheless not been evaluated.

Increased arterial stiffness has been recently shown to be related to myocardial damage in the general population [9]. Endothelial dysfunction [10] and subclinical atherosclerosis [11] may also contribute to subclinical myocardial damage. Thus the objective of this study was to determine whether arterial stiffness evaluated by brachial-arterial pulse wave velocity (ba-PWV), endothelial dysfunction measured by brachial flow mediated dilatation (FMD) and atherosclerotic burden assessed by carotid intima media thickness (IMT) are related to the myocardial injury, as indicated by high sensitivity troponin level, in patients with T2DM.

## Methods

Consecutive T2DM patients as defined by World Health Organization criteria on stable hypoglycemic and cardiovascular medication for at least 3 months were recruited from the medical outpatient clinic. Exclusion criteria included poorly controlled diabetes (hemoglobin A1c (HbA1c) ≥11%], dilated cardiomyopathy, significant valvular heart disease, chronic atrial fibrillation, New York Heart Association class III/IV heart failure, creatinine level greater than 220 mmol/liter, documented history of acute coronary syndrome, stroke, coronary revascularization and refusal to participate. The total of number of T2DM patients was 100. During the study period, 150 age- and sex-matched Chinese controls without T2DM, established cardiovascular disease or chronic inflammatory disease were recruited from a community health screening programme. Written informed consent was obtained from all study subjects. The study was approved by the local institutional review board and was conducted according to the Declaration of Helsinki.

### Clinical parameters

Baseline demographic data, clinical characteristics, blood sampling, ba-PWV, brachial FMD and carotid IMT were obtained on the same day in all study subjects. Blood pressure, body weight, body height, and body mass index (BMI) were also measured. Hypertension was defined as resting systolic or diastolic blood pressure >140 mmHg or >90 mm Hg, respectively, at two different clinic visits or the prescription of antihypertensive medication. Hypercholesterolemia was defined as fasting total plasma cholesterol ≥4.9 mmol/liter or the prescription of statins. Smoking status was recorded as ever smoker (past or current) or nonsmoker.

Serum HbA1c, total cholesterol, triglyceride, high-density lipoprotein cholesterol and low-density lipoprotein cholesterol levels, fasting glucose, HbA1c and high sensitivity C-reactive protein (hs-CRP) were measured in all subjects in a fasting venous blood sample [12]. The new serum level of high sensitivity troponin I (hs-TnI) was determined using Chemoluminescent Microparticule ImmunoAssay (Architect i1000SR Abbott®, Paris, France). The level of detection is 1.2 ng/L according to the manufacturer’s instruction and above such is considered to be a detectable hs-TnI. Prior studies demonstrated that the 99^th^ percentile of hs-TnI assays varies across different study groups [13,14]. Accordingly, the present study defined the 99th percentile based on the hs-TnI of an age-matched healthy control for both genders, respectively.

Detailed protocols for cardiovascular assessment have been previously described: (1) arterial stiffness assessed by ba-PWV (VP-2000 system; Colin Corp., San Antonio, TX, U.S.A.) [12], (2) vascular endothelial function measured as flow-mediated dilatation (FMD) of the brachial artery with a high-resolution ultrasound system (Agilent Sonons 5500; Philips, Andover, MA) [15] and, (3) carotid atherosclerosis measured as carotid IMT using a high-resolution ultrasound system (Agilent Sonons 5500; Philips, Andover, MA) [16].

### Statistical analysis

Data are expressed as mean ± standard deviation for continuous variables and frequencies or proportions for categorical variables. Continuous demographic variables of the two groups were compared using the Mann-Whitney U test and categorical demographic variables compared using Pearson Chi-square test or the Fisher’s exact test if at least one cell had an expected cell count below five. In order to detect factors associated with subclinical myocardial damage in patients with T2DM, multivariate analyses for ba-PWV, brachial FMD and carotid IMT were performed with an enter linear regression model in which each variable with P< 0.10 (according to the univariate analysis) was chosen.

All statistical analyses were performed using the statistical package SPSS for windows (Version 18.0, SPSS, Chicago, USA). All P values reported are 2-sided for consistency. A P value < 0.05 was considered statistically significant.

## Results

### Clinical characteristics

Clinical characteristics, blood tests and cardiovascular assessment of study patients is shown in Table 1. The mean duration of disease for patients with T2DM was 10.5±7.8 years and 18 (18%) were maintained on insulin therapy. Patients with T2DM had a higher systolic blood pressure, BMI, serum creatinine, fasting blood glucose, HbA1c and a lower high density lipoprotein than controls. The prevalence of hypertension and hypercholesterolemia was also higher in patients with T2DM.

**Table 1:** Clinical characteristics, blood tests and cardiovascular assessment of patients with type 2 diabetes mellitus (T2DM) and controls

**Parameters**	**T2DM**	**Controls**	**P value**
	**(n = 100)**	**(n = 150)**	
Age, years	62±10	60±9	0.20
Female, % (n)	53 (53)	51(77)	0.90
Systolic blood pressure, mmHg	138±19	121±19	<0.01
Diastolic blood pressure, mmHg	77±9	74±9	0.01
Body mass index, kg/m^2^	25.5±4.2	23.5±3.4	<0.01
**Medical history**			
Current smoker, % (n)	26 (26)	18 (27)	0.16
Hypertension, % (n)	61 (61)	11 (17)	<0.01
Hypercholesterolemia, % (n)	60 (60)	27 (40)	<0.01
**Serum lipid profile, renal function and inflammatory marker**			
Total cholesterol, mmol/L	4.9±0.9	5.1±0.8	0.15
Triglycerides, mmol/L	1.5±0.8	1.3±0.7	0.03
High density lipoprotein, mmol/L	1.4±0.4	1.5±0.4	<0.01
Low density lipoprotein, mmol/L	2.8±0.7	3.0±0.7	0.10
Creatinine, μmol/l	80±23	75±15	0.03
Fasting glucose, mmol/L	7.4±2.1	5.0±0.5	<0.01
HbA1c, %	7.6±1.2	5.9±0.4	<0.01
hs-CRP, mg/L	2.3±5.3	2.3±5.2	0.98
hs-TnI, μg/L	5.7±9.2	3.2±1.9	<0.01
Elevated hs-TnI, %(n)	10 (10)	1 (1)	<0.01
**Cardiovascular assessment**			
ba-PWV, ms^-1^	17.98±3.91	15.70±2.96	<0.01
Brachial FMD, %	2.6±3.5	5.5±4.2	<0.01
Carotid IMT, mm	0.96±0.20	0.86±0.14	<0.01

Abbreviations: *ba-PWV* brachial ankle pulse wave velocity, *FMD* flow mediated dilatation, *hs-CRP* high sensitivity C-reactive protein, *hs-TnI* high sensitivity Troponin I, *IMT* intima media thickness.

### High-sensitivity troponin I in all patients

The serum level of hs-TnI, representing the degree of myocardial injury, was significantly higher in patients with T2DM (Table 1). The concentration of hs-TnI was at or above the limit of detection in 246 patients (97%).

In this study, the 99th percentile value of serum hs-TnI level in male and female control subjects was 11.1 ng/L and 7.6 ng/L, respectively. These serum levels were defined as the cut-off values for elevated serum hs-TnI. Based on these cut-off values, patients with T2DM had a higher prevalence of elevated serum hs-TnI level than control (10% vs. 1.4%, P< 0.01).

For the whole study population, those with elevated hs-TnI were more likely to have T2DM, hypertension and a higher systolic blood pressure, creatinine and HbA1c level. Following adjustment for age, gender, T2DM, smoking history, hypertension and hypercholesterolemia, T2DM (OR = 3.44, CI = 1.03 – 12.00, P = 0.04) remained independently associated with elevated hs-TnI.

### High-sensitivity Troponin I in patients with T2DM

**Figure 1:**
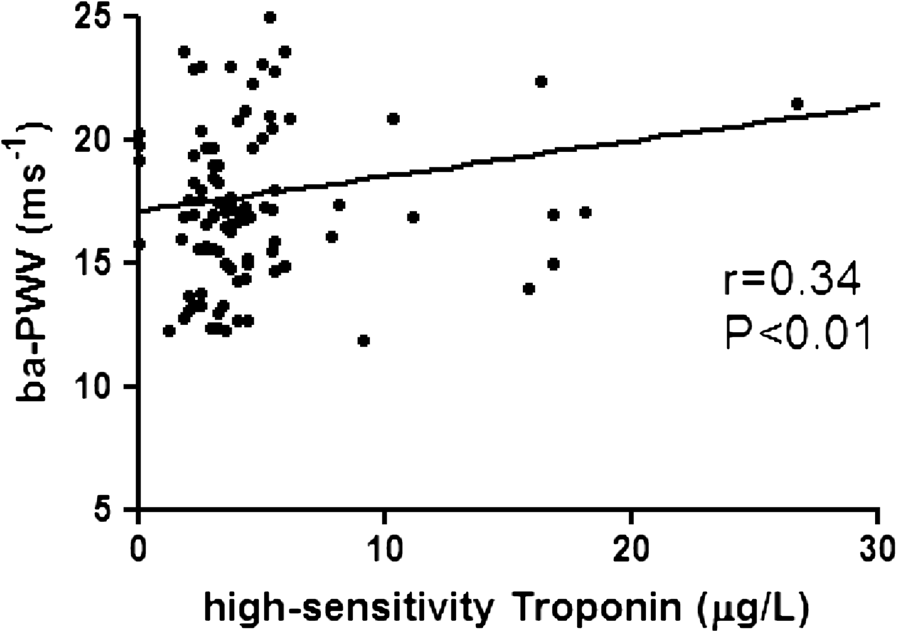
Correlation of arterial stiffness measured by brachial-ankle pulse wave velocity (ba-PWV) with high-sensitivity Troponin I.

**Figure 2:**
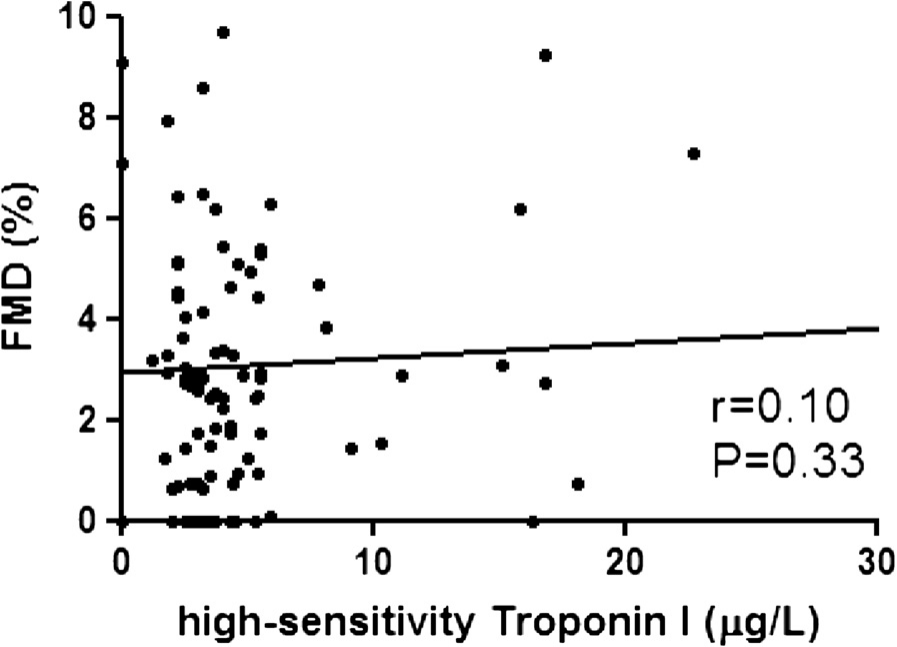
Correlation of endothelial function measured brachial flow-mediated dilatation (FMD) with high-sensitivity Troponin I.

**Figure 3:**
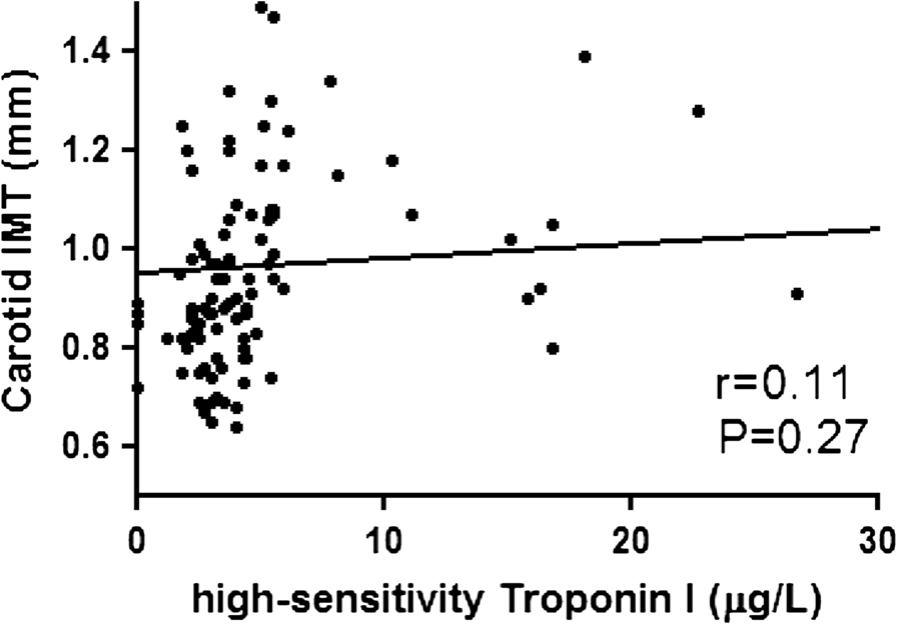
Correlation of atherosclerosis measured by carotid intima-media thickness (IMT) with high-sensitivity Troponin I.

Correlation of hs-TnI with clinical parameters in patients with T2DM is shown in Table 2. Serum level of hs-TnI was positively correlated with systolic blood pressure and serum creatinine, but not hs-CRP or duration of disease. A linear correlation of hs-TnI with cardiovascular markers in patients with T2DM is shown in Figures 1, 2, 3. The serum level of hs-TnI was positively correlated with ba-PWV, but not with brachial FMD or carotid IMT.

**Table 2:** Correlation of high-sensitivity Troponin I (hs-TnI) with clinical variables in patients with type 2 diabetes mellitus

**Clinical variables**	**hs-TnI**	
	**r**	**P value**
Age	0.08	0.46
Body mass index	0.15	0.14
Systolic blood pressure	0.31	<0.01
Diastolic blood pressure	0.12	0.26
Total cholesterol	0.05	0.66
Triglyceride	0.02	0.87
High density lipoprotein	0.05	0.60
Low density lipoprotein	0.01	0.91
Creatinine	0.26	0.01
Fasting Glucose	0.19	0.11
HbA1c	0.09	0.39
hs-CRP	0.11	0.29
Duration of disease	0.13	0.21

Abbreviations as in Table 1.

### Multivariate analysis for predictors of elevated hs-TnI in patients with T2DM

Multivariate analysis was performed to detect factors independently associated with myocardial injury measured by hs-TnI. Univariate analysis demonstrated that systolic blood pressure, serum creatinine and ba-PWV were significantly related with hs-TnI. Multivariate analysis revealed that only ba-PWV remained associated with elevated hs-TnI in patients with T2DM (Table 3).

**Table 3:** Predictors for high-sensitivity troponin I in patients with type 2 diabetes mellitus

	**Univariate analysis**			**Multivariate analysis**		
**Variables**	**β**	**95% CI**	**P value**	**β**	**95% CI**	**P value**
Age	0.08	−0.11 – 0.25	0.46			
Female Sex	0.17	−0.50 – 6.73	0.09	0.16	−1.45 – 7.44	0.18
Systolic blood pressure	0.31	0.05 – 0.24	<0.01	0.17	−0.03 – 0.19	0.16
Diastolic blood pressure	0.12	−0.10 – 0.34	0.26			
Body mass index	0.15	−0.11 – 0.76	0.14			
Current smoker	−0.03	−4.76 – 3.59	0.78			
Hypertension	0.16	−0.66 – 6.75	0.11			
Duration of disease	0.13	−0.04 – 0.20	0.21			
Total Cholesterol	0.05	−1.63 – 2.56	0.66			
Triglyceride	0.02	−2.03 – 2.41	0.87			
High density lipoprotein	0.05	−3.77 – 6.55	0.60			
Low density lipoprotein	0.01	−2.35 – 2.64	0.91			
Serum creatinine	0.26	0.02 – 0.18	0.01	0.05	−0.08 – 0.12	0.66
Fasting glucose	0.19	−0.16 – 1.92	0.11			
HbA1c	0.09	−0.83 – 2.14	0.39			
hs-CRP	0.11	−0.17 – 0.54	0.29			
ba-PWV, ms^-1^	0.34	0.30 – 1.24	<0.01	0.25	0.04 – 1.18	0.04
Brachial FMD, %	0.10	−0.27 – 0.78	0.33			
Carotid IMT, mm	0.11	−3.96 – 14.04	0.27			

Abbreviations as in Table 1.

## Discussion

The present study demonstrated that patients with T2DM have subclinical myocardial injury as detected by elevated levels of hs-TnI. Arterial stiffness evaluated by ba-PWV was the only parameter associated with hs-TnI in patients with T2DM.

Previous studies have consistently demonstrated that subclinical myocardial necrosis detected by troponin I predicted adverse cardiovascular events in patients with T2DM [17,18]. In addition to the prognostic value for future adverse cardiovascular events, the use of a high-sensitivity assay permits detection of minimally elevated troponin that represents subclinical myocardial damage [3,7,19,20]. In a recent report from the Atherosclerosis Risk in Communities (ARIC) study (n = 9661), subclinical myocardial injury, detected by high-sensitivity troponin T, was closely associated with hyperglycemia in patients with no history of atherosclerotic disease [3]. Similarly, this study demonstrates that patients with T2DM and no clinically relevant atherosclerotic disease had myocardial damage detected by hs-TnI. This suggests that T2DM contributes to subclinical myocardial injury, independent of clinically overt atherosclerotic disease.

Arterial stiffness measured by PWV (carotid to femoral) is a strong predictor for adverse cardiovascular events in community-based subjects [21]. Studies have also demonstrated that T2DM is associated with increased arterial stiffness that may partly explain the increased cardiovascular risk [22]. Although arterial stiffness has been postulated to act as a surrogate marker for underlying atherosclerosis and reflect clustering of cardiovascular risk factors, the exact mechanism of how this correlates with adverse cardiovascular outcome is uncertain. Studies have previously demonstrated that PWV was related with left ventricular diastolic dysfunction detected by echocardiography [23,24]. Further, a recent report demonstrated that increased ba-PWV was associated with high-sensitivity troponin T level in a community-based subject aged over 60 (T2DM in 26%) [9]. This suggests that arterial stiffening may cause subclinical myocardial injury, resulting in adverse cardiovascular events. The present study further demonstrated that arterial stiffness measured by ba-PWV was independently associated with elevated hs-TnI in patients with T2DM. The current results therefore suggest that increased arterial stiffness is related to subclinical myocardial injury and contribute to an adverse cardiovascular outcome in patients with T2DM.

Patients with T2DM have increased arterial stiffness due to multiple mechanisms including increased oxidative stress [25], accelerated endothelial cell apoptosis [26], endothelial dysfunction [27] and depletion of endothelial progenitor cells [28]. Arterial stiffening may subsequently cause myocardial damage in several ways although the exact mechanism is uncertain. Decreased compliance of the aorta increases systolic pressure and left ventricular preload, resulting in elevated stress on the left ventricular myocardium during the systolic phase. This may predispose patients to develop left ventricular hypertrophy that is associated with an elevated high-sensitivity troponin level [7]. Further, a reduction in diastolic pressure decreases coronary perfusion that may lead to myocardial ischemia, independent of underlying coronary atherosclerotic burden [29]. Finally, increased arterial stiffness may be indirectly related to myocardial injury via such as clustering of adverse cardiovascular risk profiles and systemic inflammation. Nonetheless the means by which arterial stiffening relates to myocardial injury in patients with T2DM requires evaluation by further prospective study.

A number of further mechanisms have also been proposed to contribute to myocardial injury in patients with T2DM. The present study thus sought to identify any such additional mechanisms. Hyperglycaemia may induce impaired brachial FMD [30], which represents an early stage of atherosclerosis. Further, carotid IMT, a well validated surrogate for subclinical atherosclerosis, has been shown to be increased in patients with T2DM and its progression can be slowed by intensive treatment [31]. The current study nonetheless demonstrated that only ba-PWV, not brachial FMD or carotid IMT, correlated with hs-TnI. A previous population-based study likewise was unable to demonstrate an association between elevated high-sensitivity troponin T level and atherosclerosis evaluated by coronary calcification [7]. These findings thus suggest that troponin release in chronic setting differs from those in acute setting, and is not associated with subclinical atherosclerosis measured by brachial FMD and carotid IMT.

### Limitations

The present study had several limitations. A causal relationship between arterial stiffness and myocardial injury could not be established because of the cross-sectional nature of the study. Although patients with T2DM were all clinically free from overt cardiovascular complications, silent coronary artery disease could not be excluded. Further, this study did not replicate the previously demonstrated association of hs-TnI with hbA1c% [3], likely due to the small study population. The possibility of residual confounding factors could not be excluded even though the results were adjusted for multivariate covariates. The use of direct assessment of atherosclerosis, such as coronary angiography or coronary endothelial function assessment using intracoronary acetylcholine infusion may provide addition information regarding the relation between atherosclerosis and hs-TnI. However, these tests are invasive in nature and therefore may not be clinically feasible to perform for research purpose. Moreover, whether advanced glycation end products, an intermediate product that is elevated in patients with T2DM and an independent marker of post-infarction heart failure, would contribute to elevation of hs-TnI would require evaluation by future studies [32].

## Conclusion

The present study demonstrated that in patients with T2DM and no clinical evidence of macrovascular disease, ba-PWV was independently associated with hs-TnI. Brachial FMD and carotid IMT showed no such association. These findings suggest that increased arterial stiffness contributes to subclinical myocardial injury in patients with T2DM, beyond the development of clinically relevant atherosclerotic disease.

## Competing interests

None of the authors have or perceived conflict of interest.

## Authors’ contributions

YK: Design of study, data collection, drafted the manuscript, ZC: Data collection, SC: Design of study, CY: Data collection, LK: Data collection, LS: Data collection, LC: Design of study, TH: Design of study, drafted the manuscript. All authors read and approved the final manuscript.
